# PROMOTING AND ATTRACTING STUDENTS TO CAREERS IN AGING SERVICES

**DOI:** 10.1093/geroni/igz038.2451

**Published:** 2019-11-08

**Authors:** Melinda Heinz

**Affiliations:** Upper Iowa University, Fayette, Iowa, United States

## Abstract

Three student focus groups were conducted at residential, center, and online university locations (N = 15) to investigate interest and understanding of careers in gerontology and exposure to aging issues. Students majoring in health services administration, psychology, and human services were recruited and given an honorarium for participating. Sessions were recorded and transcribed with two researchers independently coding to identify themes. Center and online participants were more likely to be non-traditional students. Eighty-seven percent of participants were female, 13 percent were male. Ages ranged from 19 to 34 (M = 23.4). Eighty-seven percent were upperclassmen. This study is unique as most research has investigated aging issues with traditional aged students. Center students reported aging issues were discussed in courses outside of their majors, whereas residential students stated issues did not receive attention outside of gerontology classes. Online students stated discussions depended on the class. Common deterrents for not considering careers in gerontology were concerns about performing “physical cares” or coping with death anxiety. Few considered what a career in gerontology looked like outside of nursing homes. To increase awareness, some students felt “gerontology classes should be mandatory.” Students felt taking field trips to modern nursing homes “changed their perspective,” from medical model facilities. All participants reported little exposure to older adults or gerontology as a viable career path in high school. With the rapidly aging population, we suggest incorporating a “Careers in Aging” unit in high schools to increase awareness of gerontology opportunities.

